# Do values and political attitudes affect help-seeking? Exploring reported help-seeking for mental health problems in a general population sample using a milieu framework

**DOI:** 10.1017/S2045796023000641

**Published:** 2023-08-04

**Authors:** J. Spahlholz, E. Baumann, R. Müller-Hilmer, R. Hilmer, C. Sander, S. Schindler, S. Speerforck, M. C. Angermeyer, G. Schomerus

**Affiliations:** 1 Department of Psychiatry and Psychotherapy, University of Leipzig Medical Center, Leipzig, Germany; 2 Department of Journalism and Communication Research, Hanover University of Music, Drama, and Media, Hannover, Germany; 3 pmg – Policy Matters, Political Research and Consulting mbH, Berlin, Germany; 4 Center for Public Mental Health, Gösing am Wagram, Austria

**Keywords:** mental health, mental illness stigma, population survey, psychiatric services

## Abstract

**Aims:**

Help-seeking for mental health problems is facilitated and hindered by several factors at the individual, interpersonal and community level. The most frequently researched factors contributing to differences in help-seeking behaviour are based on classical socio-demographic variables, such as age, gender and education, but explanations for the observed differences are often absent or remain vague. The present study complements traditional approaches in help-seeking research by introducing a milieu approach, focusing on values and political attitudes as a possible explanation for differences in help-seeking for emotional mental health problems.

**Methods:**

A representative cross-sectional survey of *N* = 3,042 respondents in Germany was conducted through face-to-face interviews about past help-seeking for mental health problems, socio-demographic characteristics and values and political attitudes

**Results:**

Multivariate logistic regression analyses indicated that belonging to a cosmopolitan intellectual milieu group was significantly associated with an increased likelihood of past help-seeking for mental health issues (psychotherapeutic/psychological help-seeking [OR = 2.09, 95% CI: 1.11–3.93, *p* < 0.05) and primary care (OR = 2.21, 95% CI: 1.15–4.24, *p* < 0.05]), whereas members of individualist and conservative milieu groups were less likely to report having sought help from a psychotherapist, but not from a general practitioner. Increased odds ratios were also found for a number of socio-demographic variables, such as being aged 26 years and over, a female gender and more than 12 years of formal education. Associations between socio-demographic variables remained significant, and the explained variance of the used models improved considerably when milieu variables were added.

**Conclusions:**

We discuss how milieu-specific patterns were relevant for explaining differences in mental health service use in addition to socio-demographic factors. It seems promising to consider help-seeking from a milieu perspective to improve disparities in access to and the use of psychotherapy as well as to resource allocation.

## Background

Mental disorders are highly prevalent, with a pooled lifetime prevalence of 29% worldwide (Steel *et al.*, 2014). However, even in countries with highly developed mental healthcare systems, only a low proportion of affected individuals seek professional help (Gulliver *et al.*, 2012; Wang *et al.*, 2007, 2005). For example, only 13.7% of affected adults in a German sample made treatment contact in the year of onset of any anxiety disorder and 40.4% made contact in the year of onset of any mood disorder. Across several countries, the median delay between symptom occurrence and treatment contact ranged from 3 to 30 years for anxiety disorders, 1 to 14 years for mood disorders and 6 to 18 years for substance use disorders (Wang *et al.*, 2007). The proportion of people who did not seek help remained stable over a period of 10 years in Germany (Brandstetter *et al.*, 2017). Even in the presence of considerable psychological distress and impairment due to mental disorder, and despite the availability of effective treatments, a high proportion of individuals with prevalent mental disorders remain untreated (Wang *et al.*, 2005).

Based on socio-demographic and clinical characteristics, several studies have shown that female gender, high education, increased age up to middle age and depressive symptoms in particular were associated with more frequent help-seeking (Gellert *et al.*, 2021; Rommel *et al.*, 2018; Schomerus *et al.*, 2013; von Eitzen *et al.*, 2021). At the same time, research on health inequalities demonstrated that a social gradient in self-reported mental health (MH) exists (Hapke *et al.*, 2012) and that individuals with the highest risk of mental illness (e.g., unskilled employees and low-income individuals) are less likely to use outpatient psychotherapy (Epping *et al.*, 2017).

To investigate potential explanations for the observed variations in help-seeking, stigmatizing attitudes toward mental healthcare as well as differences in knowledge and treatability of mental illnesses have been discussed (Thornicroft, 2008). Besides individual-level variables, structural barriers to help-seeking such as low healthcare density in rural areas (often accompanied with lack of transportation and long distances to accessing appropriate help) and long waiting periods before being able to start treatment have been identified as reasons for delayed help-seeking or opting not to seek psychotherapeutic treatment at all (Altmann *et al.*, 2016). Regarding psychiatric/psychotherapeutic service density, findings from Germany revealed that low healthcare density does not necessarily coincide with a lower utilization of services (Rommel *et al.*, 2018), raising the question of whether an increase in healthcare density reduces existing inequalities in the utilization of healthcare services. An approach based on the theory of planned behaviour explains help-seeking behaviour on the individual level but does little to aid our understanding of specific groups that do or do not seek help (Godin and Kok, 1996; Tomczyk *et al.*, 2020a). To overcome these limitations, the concept of milieus might be useful (Speerforck and Schomerus, 2020).

There has been a long tradition of research on social milieus in social sciences. The social milieu approach aims to identify groups of individuals with similar values, life principles, relationships and mentalities (Hradil, 2006). Following this definition, individuals in the same social milieu largely live under the same circumstances, tend to think similarly and thus tend to act the same way. Consequently, horizontal dimensions in terms of values and/or attitudes have been described as fundamental to understanding social differentiations within society while not being completely disentangled from socio-economic factors (the vertical dimension). The milieu approach offers a perspective to emphasize both vertical and horizontal dimensions (Hradil, 2006; Wippermann, 2009).

The milieu categorization used in the present study is based on findings reported by Müller-Hilmer and Gagné (2018). The authors divided a representative sample of 4,892 German adults into nine clusters using a cluster analysis based on normative values (e.g., social equity) and fundamental political attitudes (e.g., evaluations of social security along a pro-individual versus a government’s duty axis).

This socio-political approach builds on sociological milieu theory by describing heterogeneity of society with the help of political aspects. As a result, the milieus represent an intersection of basic political value orientations, everyday experiences, political attitudes and modes of political participation (Kösters and Jandura, 2019). Public discussions about concerns regarding a divided society have become more and more prominent; however, these concerns are not easily empirically confirmed (Mau, 2022). There are certainly specific issues (e.g., adequacy of climate change strategies (Salheiser *et al.*, 2022)) that ignite controversial and polarized debates. In line with this, milieu research has also identified certain topics on which opinions and viewpoints diverge considerably across different milieu groups (e.g., whether migration is an enrichment or a threat to society (Müller-Hilmer and Gagné, 2018). It is unclear whether political values and/or perceptions of polarization also extend to a person’s response to a personal MH crisis, including their readiness to seek professional help.

The purpose of this study was to examine whether self-reported help-seeking behaviour was related to being embedded in a specific milieu. Given that individuals from different milieu groups not only differ regarding their attitudes and values but also in their (inter-)actions (Hradil, 2006), we explored whether seeking psychotherapy or seeing a general practitioner (GP) for MH problems is associated with being part of a specific milieu. The milieu framework might offer a more nuanced understanding of the help-seeking process – not only from a theoretical but also from a practical point of view: Understanding the lived experiences, perceptions and needs of different milieu groups might be useful in developing strategies for meeting milieu-specific needs.

## Methods

### Participants and procedures

The survey was conducted in a representative probability sample of the German population by a market and social research institute (USUMA, Berlin, Germany). The three-stage random sampling procedure included (1) regional area sampling, (2) household selection and (3) selection of target persons within target households (ADM, 2021). People living in private households with sufficient knowledge of German and being 18 years or older were included. Due to the coronavirus disease (COVID-19), all participants were given the choice to be interviewed face-to-face or to complete the same questionnaire in paper–pencil format, while the interviewer waited outside. Face-to-face interviews were carried out following current safety regulations, wearing facial masks and keeping physical distance. From July to September 2020, data from 2,579 (84.7%) participants were collected via face-to-face interviews and data from 464 (15.3%) participants using paper–pencil format, resulting in a final sample of *N* = 3,042, reflecting a response rate of 57.1%. All participants gave informed consent orally and received written information about handling of their data and their right to withdraw from the study at any time. The questionnaire had been pretested on a random sample of *N* = 30 adults and approved by the review board of Greifswald University Medical Center (BB 195/18).

### Measures

*Milieu groups* were determined based on participants’ responses to the *value orientation scale* and the *political attitudes scale*, using discriminant functions derived from the original study by Müller-Hilmer and Gagné (2018). The *value orientation scale* is an 18-item measure designed to assess normative values or rather principles (e.g., social equity). Each 7-point Likert item is anchored at the value 1 by the label “not at all important” and the value of 7 by the label “very important”. The *political attitude scale* is a 7-point semantic differential scale comprising 28 pairs of bipolar statements on prevalent cultural, political and economic issues (Gagné *et al.*, 2018; Müller-Hilmer and Gagné, 2018), such as “The state should guarantee social security” at the left end of the scale and “The state should leave social security to the citizens’ own responsibility” at the right end. For this item, higher scores represent higher value preferences for market freedom and lower social equity preferences (Neugebauer, 2007). In order to estimate how much information each item would originally have contributed to a separation of the nine clusters of the representative sample of the German population in 2016 (Müller-Hilmer and Gagné, 2018), we calculated a discriminant analysis with the original response data and cluster solution from 2016 using the package DiscriMiner (version 0.1-29) in R version 3.6.3. This applied, the nine resulting discriminant functions would refine the original similarity-based cluster assignment of the respondents from 2016, maximizing their differences instead. We used these discriminant functions for our new respondents from 2020 to determine their cluster membership probabilities as weighted sums of their responses to the original items. Finally, a respondent’s milieu group was defined as the cluster with the highest membership probability. For further information on the original cluster analysis, see Müller-Hilmer and Gagné (2018).

*Lifetime help-seeking for MH problems with either a psychotherapist/psychologist or a GP* was assessed by asking: “Have you ever consulted a (1) psychotherapist/psychologist or a (2) primary care physician about MH problems?”. The answer format was 1 = “yes” and 2 = “no” and also offered 3 as a “no answer” category.

*Socio-demographic data* including age, gender, marital status, education and household income were assessed. Marital status was measured categorically and collapsed into three categories (single, married and widowed/divorced). Education was measured by the highest level of schooling (forced choice between nine categories; collapsed into five categories: unknown/still pupil, no schooling, 8–9 years, 10 years and 12–13 years). Household income was measured in nine categories and divided into four categories (<2,000€, 2,000€–2,500€, 2,500€–3,500€, and ≥3,500€) according to quartile distribution.

### Statistical analyses

Descriptive analyses of socio-demographic variables and help-seeking behaviour are presented as means (*M*) and standard deviations (*SD*) when continuous and as frequencies and proportions (%), when categorical. To investigate the characteristics of each milieu group, univariate ANOVAs on the clustering variables with Bonferroni-adjusted post hoc comparisons were used and compared with the characteristics of each milieu group in the original study, confirming that the socio-economic characteristics of the different milieu groups had been replicated in our sample (Müller-Hilmer and Gagné; 2018). In a further step, the labels of each milieu group were comprehensively reflected and discussed in the light of non-stigmatizing and non-discriminating language. Observed frequencies of past help-seeking were compared across milieu groups using Chi-square tests. Because of the omnibus nature of the Chi-square test, adjusted residuals (AR) were obtained for each cell to determine significant relations for past help-seeking behaviour. AR >|±2| were considered to be significant (Sharpe, 2015). Two hierarchical binary logistic regression models were performed to test the association of milieu group and self-reported help-seeking behaviour, with the participation-oriented as reference group for theoretical reasons (low educational level as well as self-ranking of social status as lowest (Müller-Hilmer and Gagné, 2018)). We controlled for age (18- to 25-year-old as reference group), gender (male as reference group), education (<12 years as reference group) and income (<2,000€ as reference group). Due to low numbers (*n* = 13), individuals who did not situate themselves within the gender binary system were excluded from the regression analyses. Multicollinearity in the predictor variables was checked before regression analyses were carried out, using the variance inflation factors (VIF). Variables with a VIF less than 10 were retained in the model. The significance level was set as *p* < 0.05, and all data analyses were conducted using Stata (16.1, StataCorp LLC, College Station, TX).

## Results

### Descriptive data

The socio-demographic characteristics are shown in Table 1. Preliminary analysis on the socio-demographics for the entire sample aged between 18 and 94 years indicated a mean age of 49.17 ± 17.35 years. 52.4% were women, and 69.1% reported more than 10 years of schooling.

**Table 1.** Sample characteristics (*N* = 3,042)

	Sample (*n* = 3,042) (%)	German general adult population^a^
Age, mean (SD)	49.17 (17.35)	–
Age groups		
18−25 years	10.4	10.4
26−45 years	32.3	29.9
46−60 years	28.5	27.3
>60 years	28.9	32.4
Gender		
Female	52.4	50.7
Male	47.2	49.3
Divers	0.4	
Education		
Unknown/pupil	1.0	0.4
No schooling completed	1.6	4.1
8−9 years	28.3	29.8
10 years	41.1	30.7
12−13 years	28.0	34.8
Income^b^		
<2,000 €	39,9	n.a.
2,000−2,500 €	16,1	n.a.
2,500−3,500 €	27,4	n.a.
≥3,500 €	16,6	n.a.

Note.

a Reference values from German Federal Statistics Office (2019).

b Comparable data on household income was not available. Total household income was assessed without taking the number of household members into account; n.a. = not available

### Milieu group characteristics (short version)

A detailed description of the milieu group characteristics, also regarding their socio-demographic characteristics, is provided in the online supplement. In brief, the milieu group labelled “committed citizenship” is characterized by a high demand for social security provided by the government and are supporting a diverse society and promoting a culture of tolerance. The “cosmopolitan intellectuals” generally express a high need for a tolerant and liberal society and stress the importance of solidarity within the European Union. “Conservatives” tend to emphasize individual performance. They value performance-related compensation and gratification (by opposing social assistance transfers and preferring a profit-oriented economy). The group of “social market optimists” shows a strong market orientation and support of a free-market economy, which they consider beneficial to all individuals; they voice a high demand for a tolerant society but are critical toward migration. People characterized as “performance-oriented” value individual performance and performance-related gratification similar to the conservatives but have a stronger preference for promotion of elites and protecting the interests of current or past high-performers, rather than addressing future generations. “Individualists” are characterized by scepticism of global social democracy, expressed by anti-migration, anti-EU and anti-globalization attitudes. The “disappointed” voice doubts and disappointment about lack of fairness, societal diversity and European solidarity. The group of “market sceptics” expresses high levels of distributional concerns and market scepticism. Finally, the “participation-oriented” report experiencing low social cohesion and express strong demands in general welfare state principles toward their own population but refuse policies related to European solidarity.

### Self-reported help-seeking behaviour

Overall, 12.8% of participants reported having consulted a GP about MH problems and 10.9% had consulted a psychotherapist. Table 2 gives the observed and the expected frequencies for each of the nine milieu groups. The results of the Chi-square test revealed that there were significant differences between the distribution of the expected and observed frequencies for consulting a psychotherapist about MH problems (*χ*^2^ (8) = 103.581; *p* < 0.001). Seeking psychotherapy was more common in the *cosmopolitan intellectual milieu group* (AR = 9.257). Being classified to the *individualist milieu group* (AR = −3.829), the *conservative milieu group* (AR = −2.694) and the *disappointed milieu group* (AR = −1.959) were associated with lower use of psychotherapy.

**Table 2.** Observed (O) and expected (E) frequencies of consulting a primary care physician and/or psychotherapist/psychologist about mental health problems

	Psychotherapist/psychologist			Primary care physician		
	O	E	AR	O	E	AR
Milieu group						
Committed citizenship (n = 426)	57	46.3	1.795	70	55.0	2.333
Cosmopolitan intellectuals (n = 355)	90	38.9	9.257	77	45.8	5.259
Conservatives (*n* = 356)	24	38.9	−2.694	33	45.7	−2.145
Social market optimists (n = 335)	30	36.7	−1.236	36	43.1	−1.225
Performance-oriented (n = 208)	20	22.5	−0.590	29	26.3	0.576
Individualists (*n* = 519)	32	56.8	−3.829	53	67.3	−2.055
Disappointed (*n* = 394)	31	42.2	−1.959	39	49.9	−1.775
Market sceptics (*n* = 332)	31	35.9	−0.915	40	42.4	−0.420
Participation-oriented (n = 117)	16	12.9	0.939	14	15.4	−0.393
Total (%)	331 (10.9)	–	–	391 (12.8)	–	–

AR = adjusted residuals. Expected frequencies were calculated by subtracting the total percentage of participants reported having consulted a psychotherapist/psychologist (10.9) or a primary care physician (12.8) from the sample size of each milieu group.

Multivariate logistic regression analysis indicated that having seen a psychotherapist/psychologist for MH problems was associated with several demographic factors (Table 3). Socio-demographic factors associated with higher odds ratios (ORs) of having seen a psychotherapist included being a woman and having higher education and higher household income. Individuals between 26 and 45 years had the highest ORs of having seen a psychotherapist. As shown in model two, adding milieu groups improved the model considerably by more than doubling the variance explained to 7.2%. The *cosmopolitan intellectuals* had nearly twofold higher ORs in self-reported help-seeking behaviour, compared with individuals from the *participation-oriented milieu group* (OR  = 2.09, 95% CI: 1.11–3.93, *p* < 0.05). In contrast, lower ORs were found for the *individualist milieu group* (OR  = 0.45, 95% CI: 0.23–0.89, *p* < 0.022) and the *conservative milieu group* (OR  = 0.48, 95% CI: 0.23–0.98, *p* < 0.045). ORs for socio-demographic factors remained significant (with exception of the age group >60 years), demonstrating the independent contribution of the milieus to the model.

**Table 3.** Determinants for psychotherapy help-seeking (*n* = 2,748): results of hierarchical binary logistic regression analysis

	Model 1		Model 2	
	OR (95% CI)	*p*-value	OR (95% CI)	*p*-value
Age groups				
18−25	R	–	R	–
26−45	2.02 (1.24−3.27)	0.004	2.15 (1.31−3.52)	0.002
46−60	1.83 (1.11−3.03)	0.018	2.00 (1.98−3.34)	0.008
>60	1.56 (0.95−2.57)	0.080	1.83 (1.10−3.06)	0.021
Gender				
Female	1.82 (1.42−2.34)	<0.001	1.74 (1.35−2.45)	<0.001
Male	R	–	R	–
Education				
<12 years	R	–	R	–
≥12 years	1.81 (1.38−2.37)	<0.001	1.45 (1.09−1.92)	0.011
Income				
<2,000 €	R	–	R	–
2,000−2,500 €	0.55 (0.38−0.80)	0.002	0.56 (0.38–0.82)	0.003
2,500−3,500 €	0.56 (0.41−0.76)	<0.001	0.62 (0.45−0.84)	0.003
≥3,500 €	0.48 (0.33−0.70)	<0.001	0.51 (0.34−0.75)	0.001
Milieu group				
Commited citizenship			0.94 (0.49−1.80)	0.862
Cosmopolitan intellectuals			2.09 (1.11−3.93)	0.022
Conservatives			0.48 (0.23−0.98)	0.045
Social market optimists			0.57 (0.28−1.15)	0.117
Performance-oriented			0.83 (0.39−1.73)	0.611
Individualists			0.45 (0.23−0.89)	0.022
Disappointed			0.54 (0.27−1.08)	0.082
Market sceptics			0.63 (0.32−1.26)	0.189
Participation-oriented			R	–
*p*-value	<0.001		<0.001	
Pseudo *R*^2^	0.033		0.072	
LR test result				
Model 1 vs. Model 2			<0.001	

Model 1 assessed the association between socio-demographic factors and help-seeking behaviour. Model 2 additionally quantified the impact of milieu group information. CI = confidence interval; OR = odds ratio; R = reference category, LR = Likelihood-ratio.

Based on the same analyses, Table 4 shows similar results regarding having seen a GP for MH problems. Seeing a GP was also associated with both socio-demographic characteristics, and milieu group membership. Being classified as a *cosmopolitan intellectual* (OR  = 2.21, 95% CI: 1.15–4.24, *p* < 0.017), as well as being a woman, and being older than 46 years significantly increased the odds of having sought help from a GP compared with those individuals of the *participation-oriented milieu group*, a male gender and age between 18 and 45 years. The total variance explained was 4.5%.

**Table 4.** Determinants for primary care help-seeking (*n* = 2,739): results of hierarchical binary logistic regression analysis

	Model 1		Model 2	
	OR (95% CI)	*p*-value	OR (95% CI)	*p*-value
Age groups				
18−25	R	–	R	–
26−45	1.65 (1.05−2.60)	0.031	1.74 (1.10−2.75)	0.018
46−60	1.57 (0.98−2.50)	0.059	1.71 (1.07−2.75)	0.026
>60	1.52 (0.96−2.41)	0.077	1.72 (1.07−2.75)	0.024
Gender				
Female	2.06 (1.63−2.60)	<0.001	2.00 (1.58−2.53)	<0.001
Male	R	–	R	–
Education				
<12 years	R	–	R	–
≥12 years	0.92 (0.70−1.20)	0.533	0.76 (0.58−1.03)	0.062
Income				
<2,000 €	R	–	R	–
2,000−2,500 €	0.61 (0.43−0.87)	0.006	0.63 (0.44−0.89)	0.009
2,500−3,500 €	0.78 (0.59−1.03)	0.086	0.83 (0.63−1.10)	0.192
≥3,500 €	0.65 (0.45−0.93)	0.019	0.68 (0.47−0.97)	0.035
Milieu group				
Committed citizenship			1.44 (0.75−2.75)	0.276
Cosmopolitan intellectuals			2.21 (1.15−4.24)	0.017
Conservatives			0.79 (0.39−1.59)	0.514
Social market optimists			0.78 (0.39−1.57)	0.485
Performance-oriented			1.33 (0.64–2.74)	0.446
Individualists			0.88 (0.45−1.69)	0.692
Disappointed			0.82 (0.41−1.61)	0.561
Market sceptics			0.89 (0.45−1.76)	0.734
Participation-oriented			R	–
*p*-value	<0.001		<0.001	
Pseudo *R*^2^	0.027		0.045	
LR test result				
Model 1 vs. Model 2			<0.001	

Model 1 assessed the association between socio-demographic factors and help-seeking behaviour. Model 2 additionally quantified the impact of milieu group information. CI = confidence interval; OR = odds ratio; R = reference category, LR = Likelihood-ratio

## Discussion

The present study examined self-reported help-seeking behaviour for MH problems focusing on milieus in a large representative population sample in Germany. We identified nine milieu groups according to Müller-Hilmer and Gagné (2018) and examined whether past help-seeking behaviour differed between these milieu groups.

The main finding of the present study is that the likelihood to seek help varies between milieus, even when controlling for known socio-demographic predictors, and that this effect was even more pronounced regarding seeking psychotherapy than seeking help from a GP. We identified one out of nine milieu groups, labelled the *cosmopolitan intellectuals*, that were more likely to have reported help-seeking both from psychotherapists and GP than other milieu groups. The *cosmopolitan intellectuals*, characterized by their liberal ideas and cultural openness, might be more open to therapeutic encounters requiring self-reflection and readiness for new experiences. Similarly to having common interests or shared activities, individuals who relate to one another communicate with others by exchanging information and expressing their ideas. Sharing experiences about MH problems, knowing about others who have been to therapists and asking them for a referral has been associated with entering psychotherapy (Kadushin, 1966). As both a cause and a consequence of help-seeking, members of the *cosmopolitan intellectual milieu group* may share knowledge, opinions and experiences about MH problems and therapy options with other group members more intensely. Consequently, feelings of being more addressed by MH services might be evoked, including the way professional help is offered (e.g., talk therapy) as well as concepts of mental illnesses (e.g., biopsychosocial model of health and disease).

Associations between specific dispositional traits of personality and political attitudes have been examined in several studies and across a range of contexts, demonstrating that openness to experiences was positively correlated with liberalism (Gerber *et al.*, 2011) and negatively to political conservatism (Osborne *et al.*, 2018). Research has further indicated that a low level of openness to new experiences and a specific set of a schema-based social worldview (e.g., that the world is unstable, insecure, dangerous and threatening) as well as specific motivational values (e.g. social control to enforce order, stability, security and traditional lifestyles) were fundamental for right-wing authoritarianism – a specific ideological attitude that constitutes the basis of social and economic conservatism (Osborne *et al.*, 2023). The *individualists* characterized by their scepticism about global social democracy, migration and globalization might fit in this pattern of a low openness to (new) experiences and a high need for security, potentially explaining why this milieu group preferred seeing a GP rather than a psychotherapist. This finding is consistent with previous research (McGowan and Midlarsky, 2012), indicating that individuals with higher authoritarian orientations were less open in disclosing their problems to MH professionals and less confident that MH professionals can help with their problems. Psychotherapists might be seen as “outsiders” (Furr and Hines-Martin, 2003), whereas GP might be seen as “insiders” due to their in-depth knowledge about the patient and their relatives, as well as their long-standing and trusting relationship.

Attitudinal barriers to psychotherapeutic help-seeking have also been identified regarding traditional masculinity norms (e.g., self-reliance, dominance, winning/competition) (Levant *et al.*, 2011). The *conservatives* characterized by their emphasis on individual performance and performance-related compensation and gratification might fit in this specific pattern of maladaptive coping strategies, such as masking symptoms or expressing them atypically (Cleary, 2012; Seidler *et al.*, 2016), and therefore increasing the risk of underdetection and undertreatment for mental illnesses (Wittchen *et al.*, 2003). Since our findings remained significant after controlling for gender, they point to the cultural and social reality of such norms beyond a person’s actual gender.

Knowledge on the objective need for psychotherapy between the milieu groups is lacking. Regarding ideas of masculinity, research has shown that traditional masculinity is an important predictor of suicidal thoughts in males (Coleman, 2015; King *et al.*, 2020; Pirkis *et al.*, 2017). Expectations to conform to traditional norms of masculinity (e.g., emotional restriction, competitiveness and aggression) further heightened the risk of suicidal behaviour in men (Coleman *et al.*, 2020). Since these norms are not restricted to men, these findings might indicate a potential high objective need for psychotherapy, particularly in the *conservatives* milieu group, which shares many of the traditionally masculine norms. Use of psychotherapy may not depend on an objective need in other milieus either.

Less educated individuals generally report a lower MH status than highly educated individuals (Hapke *et al.*, 2012), so some milieu groups (e.g., the *market sceptics* or the *disappointed*) are likely to have higher objective need, although they reported lower use of services. Likewise, there might be milieu groups (e.g., the highly educated *cosmopolitan intellectuals)* that are more knowledgeable on how to access services, regardless of an objective need. These considerations illustrate that a primary focus on the need for healthcare as seen from an external clinical or policy perspective neglects that an objective need for care does not allow any conclusion on the nature of help-seeking and a subjective need does not automatically mean that an actual need is there and that individuals seek (Bradshaw, 1972) and obtain appropriate help. Ideally, to serve all milieu groups, objective need needs to be established, people need to be ready to acknowledge their subjective need and services need to be attractive, accessible and suitable for everyone, so that individuals from all milieu groups might approach them.

Many of the described phenomena not only serve as barriers to psychotherapeutic help-seeking but also extend to therapy as the so-called “therapy interfering processes” (Seidler *et al.*, 2016). While the expression of thoughts and feelings might be not highly relevant to all milieu groups, verbal communication is important in most forms of therapy and particularly essential in traditional “talk psychotherapy” (Pascoe, 2016). In clinical practice, several preconditions are often considered to be necessary for psychotherapy (e.g., motivation for change, reflective capacities, good fit/chemistry between patient and therapist (Arndt, 2011)). The quality of the relationship between client and therapist could be influenced by several factors, including the level of similarities or dissimilarities in attitudes, values or beliefs (Marx and Spray, 1972). Considering the resourceful socio-economical background of many psychotherapists (Eller and Berg, 2023) and following the assumption that values and/or attitudes are not disentangled from socio-economic factors (Hradil, 2006), there might be several similarities with the opinions and viewpoints of the *cosmopolitan intellectuals*.

Fears of being misunderstood or being judged for the values and norms structuring the normative life-world script (Furr and Hines-Martin, 2003) might have a lower relevance for individuals belonging to the *cosmopolitan intellectual milieu group* in contrast to other milieu groups.

Taken together, these individual barriers have implications on a structural level: Psychotherapy should be open and attractive to all milieu groups, including irrespective of values, attitudes and perceptions, because otherwise the way it is provided might put certain groups that are unlikely to use it at a structural disadvantage.

### Strengths and limitations

To our knowledge, this is the first study examining reported help-seeking for MH problems using a milieu approach and nationally representative data, thus adding to our understanding of barriers and facilitators of professional help-seeking for MH problems. However, some limitations to this study need to be considered. First, no standard typology of milieus in Germany exists, and all existing typologies were limited regarding the conceptualizing of values, the consideration of socio-economic characteristics or replicability (Schröder *et al.*, 2022). For reasons of consistency and comparability, a milieu-typology already used in other research fields (Müller-Hilmer and Gagné, 2018) was chosen for the present study. Second, while milieu group membership more than doubled the variance explained by socio-demographic variables alone, the explained variance was still low at 7.2%, pointing toward other factors determining help-seeking like individual help-seeking intention or self-stigma (McLaren *et al.*, 2023; Schomerus *et al.*, 2019; Tomczyk *et al.*, 2020b). Third, self-reports on help-seeking behaviour are prone to recall bias and do not directly represent frequencies of help-seeking behaviour. Further, we asked for lifetime help-seeking regarding any lifetime MH problems but did not assess its prevalence. Accounting for temporary symptoms (e.g., during adolescence), a retrospective report of any lifetime MH problems may be prone to recall bias, influencing the validity of our results. Therefore, we solely focus on past help-seeking due to MH problems, as its recall is dependent on more severity of symptoms and impact of functioning but not on “normal” reactions to life. Finally, the probability sample of the present study proved to be representative for age, gender and region, but individuals with a higher educational level (more than 10 years of schooling) were slightly under-represented. In addition, a lack of variation in the income distribution could not be excluded because comparable data on household income for the general population was not available due to different assessment methods. Hence, we refrain from conclusions regarding the absolute prevalences of help-seeking or the size of the milieu groups but emphasize the associations between milieu groups and reported use of professional help.

## Conclusion

The findings of the present study show how using a milieu framework adds to our understanding of help-seeking for MH problems. The milieu approach offers additional findings and implications for understanding group-specific characteristics and needs, as well as identifying potential areas for interventions and future research. Beyond, it shows areas of potential structural discrimination, where services seem to meet the needs of some groups better than the needs of other groups. To shed light on milieu-specific barriers, the feeling of not being adequately addressed by the most appropriate help sources for MH problems seems to be a promising topic of future research on milieu-specific help-seeking behaviour.

## Data Availability

Data for the present study are available through the Department of Psychiatry and Psychotherapy at the University of Leipzig Medical Center, Leipzig, Germany. Contact G. Schomerus for access approval.

## Supplementary material

For supplementary material accompanying this paper visit http://doi.org/10.1017/S2045796023000641. click here to view supplementary material
