# Supplementary material for: Do values and political attitudes affect help-seeking? Exploring reported help-seeking for mental health problems in a general population sample using a milieu framework
Source: Epidemiol Psychiatr Sci. 2023 Aug 4;32:e49. doi: 10.1017/S2045796023000641 (PMC10465317; doi:10.1017/S2045796023000641)
Supplement: Supplementary file 1 [file epssup.zip › S2045796023000641sup001.docx]

# Supplementary Material

Table S1. Sample characteristics for the nine milieu groups (N=3,042)

|  | Committed citizenship  (14.0%) | Cosmopolitan intellectuals  (11.7%) | Conservatives  (11.7%) | Social market optimists.  (11.0%) | Performance-oriented  (6.8%) | Individualists  (17.1 %) | Disappointed  (13.0 %) | Market-sceptics  (10.9 %) | Participation-oriented (3.9 %) |
| --- | --- | --- | --- | --- | --- | --- | --- | --- | --- |
| **Age mean (SD)** | 45.28 (17.06) | 43.52 (16.26) | 51.70 (16.63) | 49.12 (16.74) | 49.30 (18.30) | 48.78 (17.34) | 48.00 (17.00) | 59.64 (15.78) | 48.67 (15.57) |
| 18-25 years | 13.9% | 17.2% | 5.9% | 9.0% | 11.5% | 11.8% | 10.9% | 3.0% | 5.1% |
| 26-45 years | 39.2% | 37.8% | 30.3% | 32.8% | 30.3% | 32.0% | 34.5% | 16.3% | 36.8% |
| 46-60 years | 25.4% | 27.3% | 32.3% | 31.3% | 28.9% | 27.2% | 27.9% | 27.4% | 33.3% |
| >60 years | 21.6% | 17.8% | 31.5% | 26.9% | 29.3% | 29.1% | 26.7% | 53.3% | 24.8% |
|  |  |  |  |  |  |  |  |  |  |
| **Gender** |  |  |  |  |  |  |  |  |  |
| Female | 57.8% | 56.4% | 41.0% | 61.8% | 47.6% | 47.8% | 50.8% | 57.5% | 38.5% |
| Male | 41.6% | 39.4% | 60.0% | 37.9% | 52.4% | 51.8% | 49.0% | 41.9% | 61.5% |
| Divers | 0.7% | 1.1% | 0.0% | 0.3% | 0.0% | 0.4% | 0.3% | 0.6% | 0.0% |
|  |  |  |  |  |  |  |  |  |  |
| **Marital status** |  |  |  |  |  |  |  |  |  |
| Single | 34.7% | 46.8% | 25.1% | 23.0% | 30.9% | 31.4% | 28.0% | 15.7% | 34.2% |
| Married | 44.3% | 33.8% | 52.5% | 54.6% | 47.3% | 45.7% | 51.7% | 46.4% | 45.3% |
| Widowed/divorced | 21.0% | 19.4% | 22.3% | 22.4% | 21.7% | 22.9% | 20.4% | 38.0% | 20.5% |
|  |  |  |  |  |  |  |  |  |  |
| **Education** |  |  |  |  |  |  |  |  |  |
| Unknown/pupil | 0.9% | 2.8% | 1.1% | 0.3% | 1.4% | 1.0% | 0.5% | 0.0% | 0.9% |
| No schooling completed | 1.2% | 1.1% | 0.3% | 0.6% | 6.3% | 1.5% | 2.3% | 1.2% | 2.6% |
| 8-9 years | 21.4% | 14.1% | 23.0% | 23.3% | 25.5% | 32.4% | 36.3% | 44.3% | 42.7% |
| 10 years | 36.8% | 32.4% | 45.5% | 54.9% | 39.9% | 40.5% | 39.1% | 41.0% | 41.0% |
| 12-13 years | 39.7% | 49.6% | 30.1% | 20.1% | 26.9% | 24.6% | 21.8% | 13.6% | 12.8% |
|  |  |  |  |  |  |  |  |  |  |
| **Income** |  |  |  |  |  |  |  |  |  |
| <2,000 € | 35.6% | 44.7% | 28.0% | 34.1% | 42.6% | 41.6% | 40.7% | 51.3% | 46.2% |
| 2,000–2,500 € | 14.5% | 13.7% | 11.3% | 19.0% | 21.6% | 14.3% | 18.1% | 20.1% | 17.0% |
| 2,500–3,500 € | 27.7% | 22.5% | 35.4% | 34.8% | 26.7% | 30.2% | 26.2% | 17.8% | 17.0% |
| >3,500 € | 22.14% | 19.2% | 25.3% | 12.1%% | 9.1% | 13.9% | 15.1% | 10.8% | 19.8% |
