# Supplementary material for: Do values and political attitudes affect help-seeking? Exploring reported help-seeking for mental health problems in a general population sample using a milieu framework
Source: Epidemiol Psychiatr Sci. 2023 Aug 4;32:e49. doi: 10.1017/S2045796023000641 (PMC10465317; doi:10.1017/S2045796023000641)
Supplement: Supplementary file 1 [file epssup.zip › S2045796023000641sup002.docx]

**S2 Milieu group characteristics**

The committed citizenship (n=426, 14.0%)

The committed citizenship were characterized by their high demand for a social and solidarity society (e. g. equal opportunities in educational systems (86.1%), social security as government’s duty (73.5%)). Significant mean differences in evaluations of social security along a pro-individual versus a government’s duty axis were found between all groups, except of the market-skeptic milieu group (p<.001). There was a high degree of tolerance in general, and tolerance toward homosexuality, and migration in particular (e. g. feeling foreign in their own country due to immigration (13.1%), attributing a particular self-responsibility for welcoming refugees (50.0%), more political engagement for equal rights to individuals who are homosexual (74.8%), gender equality in job, household and childcare (75.2%). Significant mean differences were revealed in items related to migration, as well as gender and sexual equality, with the committed citizenship being the most tolerant to sexual equality (p<.001), and to migration as well as gender equality along with the cosmopolitan intellectual milieu group (p<.001).

The cosmopolitan intellectuals (n=355, 11.7%)

The cosmopolitan intellectual milieu group, was named in the light of its high need for a tolerant and modern society and its high degree of openness to migration (e. g. more political engagement for equal rights to individuals who are homosexual (89.4%), gender equality in job, household, and childcare (85.3%), regarding refugee movements as an enrichment for society (71.2%), attributing a particular self-responsibility for welcoming refugees (73.0%)). There were significant mean differences in items related to migration and European solidarity between the nine milieu groups, with the cosmopolitans being the most tolerant to migration (p<.001). Significant mean differences in evaluations of European solidarity along a pro-EU versus an anti-EU policy axis were found between all groups (p<.001), with the cosmopolitans being the most tolerant (e. g. supporting decision-making on EU-level (51.0%), support of EU-member states in case of need (69.6%)).

The conservatives (n=356, 11.7%)

The conservative milieu group was characterized by its middle to low demand for a social and solidarity society (e. g. social security of the population (41.4%), and acceptance of compensatory practices (22.1%)). The highest demand for avoidance of social assistance transfers (58.2%) was found in this group (p<.001) and the importance of individual performance (48.9%) versus solidarity behavior (18.2%) was rated highest (p<.001). The highest demand for a profit-oriented economy (53.9%) versus an economy for the common good was found in this group (18.0%) (p<.001).

The social market optimists (n=335, 11.0%)

The social market optimists, were characterized by its strong market orientation (e. g. preferring an economy where the government sets up no regulations (56.1%), regarding globalization (55.2 %) as strategy for generating jobs), as well as preferring an economy for the common good (68.3%)). The market optimists generally expressed less concerns about a free market economy (p<0.01). Across the nine groups, significant mean differences in evaluations of achieving occupational success were found (p<.001), with the social market optimists emphasizing individual performance (65.4%) in contrast to socio-cultural background (17.3%). There was a high need for a tolerant society (e. g. more political engagement for equal rights to individuals who are homosexual (85.6%) and gender equality in job, household, and childcare (85.7%)), but critics against migration (e. g. attributing a particular self-responsibility for welcoming refugees (25.5%)). Significant mean differences in evaluations of equality of men and women were found between all groups (p<.001), with the social market optimists having the highest mean scores (M=6.2).

The performance-oriented (n=208, 6.8%)

The performance-oriented milieu group consists of individuals that have a low demand for a social and solidarity society, while simultaneous supporting individual self-responsibility (e. g. equal opportunities in educational systems (21.2%), acceptance of compensatory practices (28.9%), self-responsibility in social security (40.3%)). Significant mean differences in evaluations of educational equity were found between all groups (p<.001), with the performance oriented affirming an education policy that brings out the best in oneself (53.4%) in contrast to educational equity (21.2%). The idea, that social security is primarily a government’s duty was affirmed by the minority (29.8%). There were significant mean differences between the performance-oriented (high individual responsibility) and all milieu groups (social security as government’s duty), except of the conservatives (p<.001). The need to protect the interests of individuals that already improved well-being of society was affirmed more often (40.9%) by the performance-oriented (p<0.001), whereas a politics of policymaking addressing future generations was affirmed by the minority (20.7%).

The individualists (n=519, 17.1%)

The individualists were named due to its middle demand for a social and solidarity society (e. g. equal opportunities in educational systems (58.0%), acceptance of compensatory practices (52.4%)). Significant mean differences in evaluations of educational equity were found between all groups (p<.001), with the individualists affirming educational equity (58.0%) in contrast to an education policy that brings out the best in oneself (14.6%). There was a low need for a tolerant and modern society (e. g. more political engagement for equal rights to individuals who are homosexual (39.5%), female’s childcare and household responsibilities (39.5%)), and their concerns focused on migration and trade politics within the EU (e. g. feeling foreign in their own country due to immigration (40.5%), attributing a particular self-responsibility for welcoming refugees (27.6%), supporting decision-making on EU-level (47.2%)).

The disappointed (n=394, 13.0%)

The disappointed milieu group was named due to its mismatch between current state of the society and future demands. There was a strong demand in equal opportunities in educational systems (88.4%), while assuming a relationship between personal development and socio-cultural background (71.8%). Across the nine groups, significant mean differences in evaluations of achieving occupational success were found (p<.001). There were significant mean differences in evaluations of society, with the disappointed evaluating the middle of society as most disappearing or declining (82.2%), with except of the responsibles (p<.001). There was a middle need for a tolerant society (e. g. more political engagement for equal rights to individuals who are homosexual (51.8%), and gender equality in job, household, and childcare (54.6%)). Significant mean differences in evaluations of equality of men and women were found between all groups (p<.001), with the disappointed having the lowest mean scores (M=3.3). Significant mean differences in evaluations of European solidarity along a pro-EU versus an anti-EU policy axis were found between all groups, except of the participation-oriented milieu group (p<.001), with the disappointed refusing a politics of European solidarity (e. g. too many decision-making on EU-level (66.0%)).

The market-sceptics (n=332, 10.9%)

The market-sceptics milieu group was named due to its high level of distributional concerns and market skepticism (e. g. regarding globalization (63.0 %) and the TTIP agreement between the EU and the US (58.3%) as synonymous to job losses). There were significant mean differences in items related to competition and open markets between the nine milieu groups, with the market-sceptics having the highest level of concerns (p<.001). Status quo of society was evaluated as predominant unequal (e. g. there is no middle of society (81.3%)) and the level of social cohesion was rated low (69.2%). Significant mean differences in evaluations of social cohesion were found between all groups, except of the conservatives (p<.001).

The participation-oriented (n=117, 3.9%)

The participation-oriented milieu group by comparison had low mean scores due to perceived status quo of society. Status quo of society was evaluated as predominant unequal and separated (e. g. there is no middle of society (75.2%), the level of social cohesion is low (77.8%)). Significant mean differences in evaluations of social cohesion were found between all groups (p<.001). There was a strong demand in general welfare state principles of the own population (e. g. social security of the population (83.3%), acceptance of social transfers (71.8%)), but refusing a politics of European solidarity (e. g. too many decision-making on EU-level (70.5%)), and pro-migration (e. g. feeling foreign in their own country due to immigration (91.4%), rejecting refugees to avoid an overload of the social system (94.0%)).
